# Reduced Syntactic Processing Efficiency in Older Adults During Sentence Comprehension

**DOI:** 10.3389/fpsyg.2018.00243

**Published:** 2018-03-01

**Authors:** Zude Zhu, Xiaopu Hou, Yiming Yang

**Affiliations:** ^1^School of Linguistic Sciences and Arts, Jiangsu Normal University, Xuzhou, China; ^2^Jiangsu Collaborative Innovation Center for Language Competence, Xuzhou, China; ^3^Jiangsu Key Laboratory of Language and Cognitive Neuroscience, Xuzhou, China; ^4^Institute of Linguistic Sciences, Jiangsu Normal University, Xuzhou, China

**Keywords:** aging, ERP, P600, syntactic processing, neural efficiency

## Abstract

Researchers have frequently reported an age-related decline in semantic processing during sentence comprehension. However, it remains unclear whether syntactic processing also declines or whether it remains constant as people age. In the present study, 26 younger adults and 20 older adults were recruited and matched in terms of working memory, general intelligence, verbal intelligence and fluency. They were then asked to make semantic acceptability judgments while completing a Chinese sentence reading task. The behavioral results revealed that the older adults had significantly lower accuracy on measures of semantic and syntactic processing compared to younger adults. Event-related potential (ERP) results showed that during semantic processing, older adults had a significantly reduced amplitude and delayed peak latency of the N400 compared to the younger adults. During syntactic processing, older adults also showed delayed peak latency of the P600 relative to younger adults. Moreover, while P600 amplitude was comparable between the two age groups, larger P600 amplitude was associated with worse performance only in the older adults. Together, the behavioral and ERP data suggest that there is an age-related decline in both semantic and syntactic processing, with a trend toward lower efficiency in syntactic ability.

## Introduction

Sentence comprehension is one of the key components of human language. In order to construct a meaningful representation of a given sentence, one has to recognize single words and integrate word-level semantic blocks into a larger semantic utterance under the guidelines of semantic and syntactic rules (Hagoort, 2005, 2013). It remains unclear whether and how these semantic and syntactic processes decline in advanced aging.

With age, older adults have a larger vocabulary (Park and Reuter-Lorenz, 2009) and greater language expertise (reviewed in Diaz et al., 2016), and show an advantage over younger adults in building mental representations (Madden and Dijkstra, 2010). Nevertheless, behavioral and neural results have frequently shown an age-related decline during semantic processing (reviewed in Wlotko et al., 2010). One important neural component linked with semantic processing in event-related potential (ERP) studies is the N400, which peaks at about 400 ms after the word onset. Additionally, in fMRI studies, semantic integration is associated with activation in the left frontal-temporal network (Hagoort et al., 2009). In older adults, however, the N400 amplitude is significantly reduced (Federmeier et al., 2010; Wlotko et al., 2010; Xu et al., 2017). In addition, among older adults activity in the left frontal-temporal network during semantic processing is lower in poorer readers than in better readers (Grossman et al., 2002b). Activity in the left frontal-temporal network is also more reduced in older adults than younger adults during auditory sentence comprehension tasks (Peelle et al., 2011; Tremblay et al., 2013). The aging brain tries to adaptively compensate for the decline in semantic ability with predictive strategies (Federmeier, 2007) and enhanced activation in regions within and beyond the language network (Peelle et al., 2010; Diaz et al., 2016; Du et al., 2016).

While research has documented this age-related decline in semantic processing, there is still debate concerning whether syntactic processing also declines during aging. On the one hand, a series of cross-sectional and longitudinal behavioral studies have shown that older adults show impairment in certain syntactic constructions during comprehension and production (Kemper, 1986; Kemtes and Kemper, 1997; Kemper and Herman, 2006; Peelle et al., 2010). On the other hand, it has also been reported that younger and older adults show comparable syntactic processing, seen in behavioral performance (Tyler et al., 2010; Campbell et al., 2016), brain evoked responses (Kemmer et al., 2004) and brain functional networks (Campbell et al., 2016).

One challenge has to do with the criteria for identifying decline or preservation of syntactic processing. When older adults show worse behavioral performance than younger adults, it is easy to infer a decline in syntactic processing during aging. However, if younger and older adults show comparable performance, it is more difficult to deduce the reason. One possibility is that the older adults preserve the neural system and therefore show preserved performance (Campbell et al., 2016); another explanation is that older adults utilize a compensatory mechanism to maintain their performance (Cabeza et al., 2002; Davis et al., 2008; Park and Reuter-Lorenz, 2009). Given this debate, it is critical to test the functional significance of the brain response alteration in aging (Cabeza and Dennis, 2012). For instance, increased brain response should be attributed to successful compensation if the response is positively correlated with behavioral performance (Cabeza and Dennis, 2012; Hakun et al., 2015).

Another challenge in this area is that performance on syntactic processing tasks is typically conflated with other factors, such as working memory and semantic processing. For example, syntactic complexity has been manipulated by asking participants to read complex sentences vs. simple sentences (Grossman et al., 2002a). However, reading syntactically complex sentences may overload working memory, which is known to decline during aging (Kemtes and Kemper, 1997; Park and Reuter-Lorenz, 2009), and this overload may fully mediate age-related changes in syntactic ability (Waters and Caplan, 2005). Critically, however, working memory capacity has rarely been controlled in these studies. Furthermore, unlike morphological syntactic violations, which are thought to be independent of semantics, sentences with complex syntax make it difficult to integrate semantic information. Therefore, when there is an observed decline in syntactic processing it remains unclear whether the decline is due to syntactic processing *per se* or to confounding factors.

The aim of the present study was to test whether syntactic processing, in addition to semantic processing, declines during aging. To control for the confounding effects of other cognitive skills, the younger and older adults were well matched on working memory capacity, general intelligence, verbal intelligence, and verbal fluency. To better isolate the syntactic effect, we employed the violation paradigm in which congruent sentences (CON), sentences with semantic violation (SEM), and sentences with both semantic and syntactic violation (SEM + SYN) were included. The reason for including SEM + SYN sentences rather than sentences containing only syntactic violation is that syntactic violation always accompanies semantic violation in Chinese. Unlike in Indo-European languages, there is very little explicit morphology in Chinese (e.g., no inflectional indicators or case markers). The lexical category of Chinese words is often inconsistent with their syntactic function. Apart from this, there are also no intra-sentence concordance rules in Chinese. Therefore, the syntactic status of Chinese words relies highly on the context. As a result, the semantic information of these words is often used to identify their grammatical consistency. Although the syntactic violation accompanies semantic violation, the contrast between SEM + SYN and SEM could help to separate the syntactic effect from the semantic effect (Wang et al., 2008; Zhang et al., 2013).

A further feature of the present study is that we employed the ERP technique. An advantage of using ERPs is that syntactic processing has been associated with specific ERP components (Friederici, 2002). One key component is the P600 (Osterhout and Holcomb, 1992; Hagoort et al., 1993), which is a positive deflection at centro-parietal electrodes that typically starts 500–600 ms after critical word onset. Syntactic processing in Chinese has also been associated with the P600 effect (Yu and Zhang, 2008; Wang et al., 2012). Chinese BA and BEI constructions, which have been commonly used in research (Zhang et al., 2013; Yang et al., 2015) represent SVO and OSV sentences respectively. The BA and BEI constructions are specific structures, in which the object and subject noun phrase (NP) occur before the verb but after the BA or BEI marker, respectively. If the expected object NP or subject NP in BA and BEI sentences is replaced by a verb, a larger P600 would be found, relative to the P600 if the expected word had indeed been an NP (Zhang et al., 2013; Yang et al., 2015). Thus, the ERPs could further help us to test this specific syntactic effect.

The behavioral response, especially the difference in accuracy between the SEM and the SEM + SYN condition, could determine if syntactic processing declines during aging. Moreover, the correlation between the ERP data and behavioral data (i.e., accuracy) could help us to determine the functional significance of the P600 effect. According to a recent proposal (Cabeza and Dennis, 2012), compensation is indicated by an increase in neural response accompanied by higher accuracy. By contrast, increased neural response accompanied by worse performance in older adults suggests less efficient response in the older adults than the younger adults (Zhu et al., 2015).

## Materials and Methods

### Participants

Twenty-six younger adults (9 males, 18–27 years of age, *M* ±*SD* = 21.9 ± 2.5 years,) and 20 healthy older adults (11 males, 56–79 years of age, 67.6 ± 5.9 years) were recruited from the local community to participate in the experiment. All were right-handed, native Chinese speakers with normal or corrected-to-normal vision. None had a history of reading disabilities or neurological/psychiatric disorders. All the participants gave their written informed consent to participate in the experiment. While the two groups had a comparable gender ratio (*x*^2^ = 1.91, *p* = 0.17), the younger adults had more years of education (15.6 ± 1.5 years) than the older adults [13.9 ± 2.1 years; *t*(44) = 3.28, *p* = 0.02]. Therefore, years of education was controlled for in the ERPs and behavioral analyses.

### Neuropsychological Tests

In addition to a test of general cognitive status, several neuropsychological tests were administered to match younger and older adults. The measure of general cognitive status was the Beijing version of the Montreal Cognitive Assessment (MoCA)^1^, which was administered to the older adults to test their short-term memory recall, visuospatial skill, executive functioning, language, orientation, attention, concentration, and working memory. The mean score on the MoCA was 27.4 (*SD* = 1.7, range 24–30), indicating that the older participants were all cognitively healthy.

The Raven’s Standard Progressive Matrices (RSPM) was administered to test participants’ general intelligence. The RSPM score was comparable between younger adults (108.2 ± 15.1) and older adults [108.9 ± 14.1; *t*(30) = 0.06, *p* = 0.76].

The verbal subtests of the Wechsler Adult Intelligence Scale-Revised by China (WAIS-RC; Gong, 1992) were administered to measure participants’ verbal intelligence. The score on each subtest was standardized and a participant’s standardized subtest scores were summed to create their final score for verbal intelligence. The verbal intelligence score was comparable between younger adults (79.0 ± 6.0) and older adults [78.1 ± 10.2; *t*(30) = -0.32, *p* = 0.75].

In addition, following the reading span test (Daneman and Carpenter, 1980), a verbal working memory (VWM) test was administered for each participant. In the VWM test, the participants were asked to read aloud a block of sentences and recall the last word in each sentence at the end of each block. In a block, the number of sentences varied from 2 to 7. The total number of recalled words served as the VWM score. The results revealed comparable VWM scores between the younger adults (20.6 ± 3.9) and the older adults [22.2 ± 4.5; *t*(44) = 1.31, *p* = 0.20].

Furthermore, verbal fluency, which has been associated with the N400 effect in the previous literature (Federmeier et al., 2010), was measured for each participant. The verbal fluency test was administered by asking the participant to generate as many names of animals and tools as possible in 1 min, to assess animal fluency and tool fluency respectively. The total number of animal and tool names generated was comparable between younger adults (44.4 ± 11.5) and older adults [48.5 ± 3.6; *t*(44) = 1.55, *p* = 0.13].

### Stimuli

A total of 120 sentences sets were constructed for the experiment. The three sentences in each set represented all of the following three types: Congruent sentences (the CON condition), sentences with a semantic violation (the SEM condition), and sentences with combined semantic and syntactic violations (the SEM+SYN condition). As illustrated in **Table 1**, in the SEM condition, the critical verb (clean/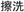) in the CON condition was replaced by another verb, which semantically mismatched the context. In the SEM + SYN condition, the critical verb in the CON condition was replaced by a noun, which semantically and syntactically mismatched the context. The critical words were the only difference among conditions. Across the three conditions, the critical words were matched for word frequency [2.43 ± 0.61, 2.43 ± 0.59, 2.55 ± 0.63, for CON, SEM, and SEM + SYN respectively; *F*(2,238) = 1.57, *p* = 0.21] and number of strokes in the Chinese characters [17.1 ± 3.87, 17.2 ± 4.01, 16.3 ± 3.55, for CON, SEM, and SEM + SYN respectively; *F*(2,238) = 2.05, *p* = 0.13].

**Table 1:** Sentence examples for each of the three experimental conditions.

Condition	Example
CON	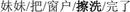
	The younger sister **cleaned** up the window.
SEM	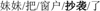
	The younger sister **plagiarized** the window.
SEM + SYN	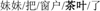
	The younger sister **tea** the window.

*Examples are given in Chinese with English translations. The critical words are in bold.*

An independent group of participants (*n* = 30) rated the semantic acceptability of the sentences on a 5-point Likert scale from 1 (fully unacceptable) to 5 (fully acceptable). The three conditions showed significant differences [*F*(2,238) = 1127.65, *p* < 0.001]. Consistent with the purpose of the manipulation, the two incongruent conditions (SEM: 1.82 ± 1.03; SEM + SYN: 1.69 ± 1.03) were both rated as significantly less acceptable than the congruent (CON: 4.79 ± 0.45) condition (*p*s < 0.001), and there was no significant difference between the two incongruent conditions (*p* = 0.68).

The sentences were divided into three lists, counterbalanced across conditions. Each list contained 40 sentences for each condition, making a total of 120 sentences. Only one sentence from each set of three sentences appeared in each list. The order of the sentences was randomized for each list and then presented in the same order to all participants. Each participant viewed only one list.

### Procedure

Participants were seated in a comfortable chair approximately one meter away from a computer screen in a sound-attenuating, electrically shielded room. All sentences were presented word-by-word at the center of the screen using E-prime software (2.0), and each word consisted of no more than three Chinese characters. Participants’ brainwaves were recorded while reading the sentences. For each trial, after a 300 ms fixation point the sentence was presented word-by-word, with 500 ms for each word, followed by a 100-ms blank screen at the end of the sentence. Then a question mark was presented for 3000 ms, during which the participants were asked press a button as accurately as possible to indicate whether the sentence was plausible or not. A blank screen was then presented for 1500 ms before the next trial began. A brief practice session was conducted to make sure that participants were familiar with the procedure. During the recording, they were asked to remain still and to avoid blinking while the stimuli were being presented.

### EEG Recording and Preprocessing

The electroencephalogram (EEG) was recorded from 62 Ag/AgCl electrodes attached to an elastic cap (Quick-Cap, Neuroscan Inc., United States). The EEG was recorded continuously with a 70 Hz low-pass filter sampled at 500 Hz, with 10 s constant time. All electrode impedances were kept below 5 kΩ before recording. The EEG data were re-referenced off-line to the average of the two mastoids and then filtered with a 0.1–30 Hz bandpass filter. The horizontal and vertical electrooculograms were also recorded. Critical epochs ranged from -200 to 1000 ms relative to the onset of the target word, with -200 to 0 ms serving as the baseline. The artifact rejection criterion was ±90 μV. Epochs that included eye blinks were removed by visual inspection. Less than 5% of trials were rejected, and the rejection rate was comparable among the three conditions. The ERP data were then averaged for each participant and each condition for correct trials only.

### Statistics

According to the experimental design and different time course among conditions, the semantic effect was examined by contrasting ERP data in the SEM and CON conditions, and the syntactic effect was examined by contrasting ERP data in the SEM + SYN and SEM conditions. For both analyses, the N400 and P600 effects were examined. First, from visual inspection, the time courses of the N400 and the P600 effects were delayed in the older adults compared to the younger adults. Moreover, as the N400 amplitude differences across conditions may influence the amplitude of the P600, we followed previous research (Hagoort, 2003) and made a re-normalization correction for the P600 by using the N400 time window as baseline.

Peak latency was assessed following procedures reported in previous studies (Federmeier and Kutas, 2005; Federmeier et al., 2010). Peak latency of the semantic effect was measured by the difference in waveforms between the SEM and CON conditions, and peak latency of the syntactic effect was measured by a similar comparison of the SYN + SEM and SEM conditions. For the semantic effect, the time point showing the largest effect was set as the peak latency for N400 and P600 in a window of 250–600 ms and 600–900 ms respectively. For the syntactic effect, the time point showing the largest effect was set as the peak latency for N400 and P600 in a window of 250–500 ms and 400–800 ms respectively.

The N400 and P600 mean amplitudes were assessed in a 200 ms window around the peak latency for each group (Kutas and Iragui, 1998; Federmeier et al., 2010; Lacombe et al., 2015). In the contrast between the SEM and CON conditions, the time window for the N400 effect was 300–500 ms for younger adults and 350–550 ms for older adults, and the time window for the P600 effect was 600–800 ms for both groups. In the contrast between the SEM + SYN and SEM conditions, the time window for the N400 effect was 250–400 ms for younger adults and 300–500 ms for older adults, and the time window for the P600 effect was 430–630 ms for younger adults and 530–730 ms for older adults.

For peak latency and amplitude analyses, the electrodes were grouped as anterior and posterior regions in the left and right hemispheres (F1, F3, F5, FC1, FC3, and FC5 for left anterior; F2, F4, F6, FC2, FC4, and FC6 for right anterior; P1, P3, P5, CP1, CP3, and CP5 for left posterior; and P2, P4, P6, CP2, CP4, and CP6 for right posterior). Repeated-measures ANCOVAs were then performed on the averaged data with Group (Younger, Older) as a between-subjects factor; Condition (CON, SEM, SEM + SYN), Hemisphere (Left, Right), and Region (Anterior, Posterior) as within-subjects factors; and years of education as a covariate. The Condition factor was not included in peak latency analysis as peak latency assessment was based on difference waveforms between conditions. Simple main effects were tested if there was a significant interaction involving Group or Condition, with Bonferroni correction used for multiple comparisons. The Greenhouse–Geisser correction was applied when evaluating effects with more than one degree of freedom to protect against Type I errors from sphericity violations.

To evaluate the functional significance of the ERP change, we tested whether the amplitude of the ERP component was correlated with the semantic (or syntactic) effect, defined in terms of accuracy. Only ERP data from the posterior region were used, as both the N400 and P600 were centro-parietally distributed. As the semantic effect could be reflected in both N400 and P600 time windows, both N400 and P600 were used as the ERP components in calculating the correlation between the ERP data and the semantic effect. For the syntactic effect, we mainly focused on the syntactic related P600 time window as the P600 is the main index for syntactic processing in BA and BEI constructions (Zhang et al., 2013). First, overall correlations were calculated to represent the association between the amplitude difference in the N400 and P600 time windows and accuracy difference for the semantic effect (SEM - CON). For the syntactic effect, we correlated the amplitude difference in the P600 time window and the accuracy difference between SEM + SYN and SEM conditions. Both correlations were performed in the group of older adults and in the group of younger adults. Then the behavioral-ERP correlation analyses were conducted within each condition (CON, SEM, and SEM + SYN) in each group.

## Results

### Behavioral Results

For the behavioral results, only accuracy (and not reaction time) was included in the analyses, as the response was made after the target word presentation. The mean accuracy in the CON, SEM, and SEM + SYN conditions was 98.0 ± 2.8%, 99.7 ± 1.1%, and 98.0 ± 1.1% in the younger adults respectively, and 88.2 ± 13.1%, 85.2 ± 14.3%, and 92.3 ± 12.5% in the older adults respectively. The result revealed a significant Group effect [*F*(2,84) = 12.72, *p* = 0.001] and a Group by Condition interaction [*F*(2,84) = 7.05, *p* = 0.001], but no Condition effect (*F* < 1). Compared to the younger adults, older adults showed significantly lower accuracy in the CON (*p* = 0.006) and SEM (*p* < 0.001) conditions, and a trend toward lower accuracy in the SEM + SYN condition (*p* = 0.06). In younger adults, there was no significant difference among conditions (*ps* > 0.37). In older adults, accuracy in the SEM + SYN was significantly higher than that in the SEM condition (*p* = 0.001), and there was a trend toward a significant difference between the CON and SEM conditions (*p* = 0.08).

### ERP Results

**FIGURE 1:**
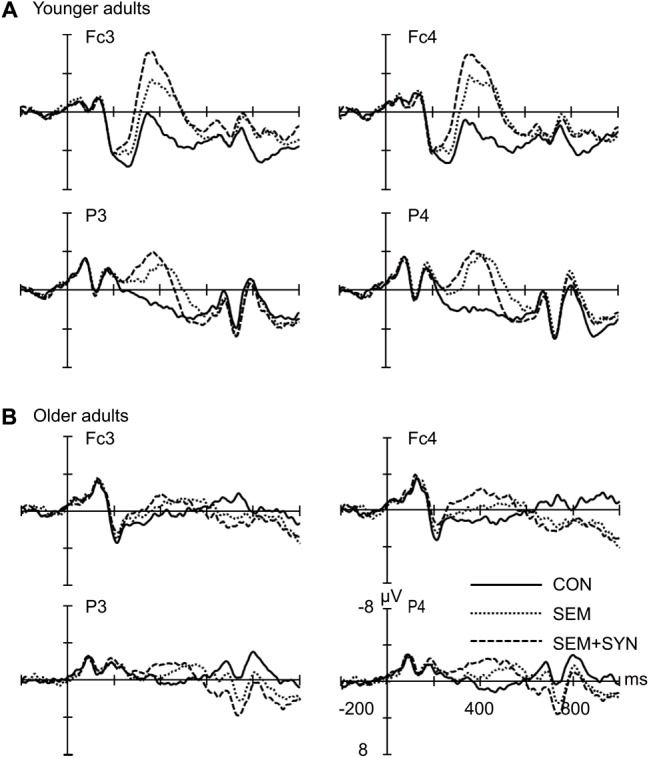
Grand averaged ERPs evoked by the critical words at four representative electrodes. **(A)** Grand averaged ERPs in younger adults. **(B)** Grand averaged ERPs in older adults. The solid line represents congruent sentences (CON), the dashed line represents sentences with semantic violation (SEM), and the broken line represents sentences with both semantic and syntactic violations (SEM + SYN). Waveforms are time-locked to the onset of the critical words. Negative is plotted upward.

**FIGURE 2:**
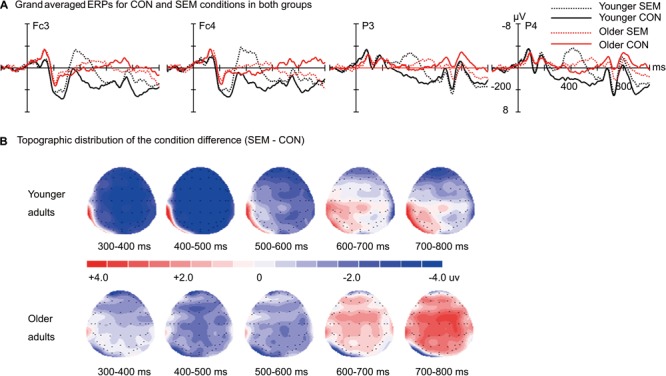
Semantic effect in younger and older groups. **(A)** Grand averaged ERPs evoked by the critical words in CON and sentences with semantic violation (SEM). **(B)** Topographic distribution of the semantic effect (SEM – CON) in younger and older groups from 300 to 800 ms after the critical word onset. Waveforms are time-locked to the onset of the critical words. Negative is plotted upward.

**FIGURE 3:**
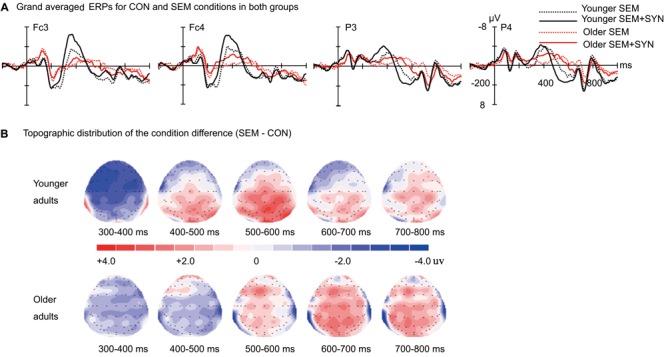
Syntactic effect in younger and older groups. **(A)** Grand averaged ERPs evoked by the critical words in sentences with semantic violation (SEM) and sentences with semantic and syntactic violation (SEM + SYN). **(B)** Topographic distribution of the semantic effect (SEM + SYN – SEM) in younger and older groups from 300 to 800 ms after the critical word onset. Waveforms are time-locked to the onset of the critical words. Negative is plotted upward.

The time courses of the ERP responses in the three experimental conditions are illustrated in **Figure 1** for younger and older adults. As revealed in the time course and topographic maps for the semantic effect (SEM - CON; **Figure 2**) and syntactic effect (SEM + SYN - SEM; **Figure 3**), the peak in the N400 window for the semantic effect and the peak in the N400 and P600 windows for the syntactic effect were delayed in the older adults relative to the younger adults.

In the semantic analysis, the mean peak latency of the N400 was significantly delayed in the older adults (472 ms) compared to the younger adults [420 ms; *F*(1,43) = 21.79, *p* < 0.001]. There was a significant Region by Group interaction [*F*(1,43) = 5.79, *p* < 0.001], revealing delayed latency in older adults compared to younger adults in both anterior and posterior regions. The mean peak latency of the P600 in the older adults (730 ms) was also significantly delayed compared to that of the younger adults (691 ms), and this was true both with [*F*(1,42) = 4.15, *p* < 0.05] and without controlling for the N400 delay [*F*(1,43) = 11.59, *p* = 0.001].

In the syntactic analysis, the mean peak latency of the N400 was significantly delayed in the older adults (395 ms) compared to the younger adults [334 ms; *F*(1,43) = 31.07, *p* < 0.001]. There was a significant Region by Group interaction [*F*(1,43) = 5.79, *p* < 0.001], revealing delayed latency in older adults compared to younger adults in both anterior and posterior regions. The mean peak latency of the P600 in the older adults (638 ms) was also significantly delayed compared to that of the younger adults (525 ms), and this was true both with [*F*(1,42) = 22.72, *p* < 0.001] and without controlling for the N400 delay [*F*(1,43) = 64.22, *p* < 0.001].

### Semantic Effect

The statistical results regarding amplitude differences across age groups and experimental conditions are summarized in **Table 2**. In the N400 time window, the global ANCOVA showed a marginally significant main effect of Condition [*F*(1,43) = 3.03, *p* = 0.09], but no significant main effect of Group (*F* < 1). However, there was a significant Group by Condition interaction [*F*(1,43) = 13.83, *p* < 0.001]. Simple main effects revealed that the N400 amplitude in the SEM condition was larger than that in the CON condition in both younger (*p* < 0.001) and older (*p* = 0.03) adults, whereas no significant group difference was found in either the SEM (*p* = 0.12) or CON (*p* = 0.27) conditions, suggesting that the interaction was due to the N400 effect being smaller in the older adults compared to the younger adults. In addition, there was a significant Condition by Region by Hemisphere by Group interaction [*F*(1,43) = 5.13, *p* < 0.05], a significant Condition by Region by Hemisphere interaction [*F*(1,43) = 6.26, *p* < 0.05], and a marginally significant Condition by Region interaction [*F*(1,43) = 3.47, *p* = 0.07]. Simple main effects revealed significantly higher N400 amplitude in the SEM condition compared to the CON condition in anterior and posterior regions in both the left and right hemisphere for younger adults (*ps* < 0.01), whereas the same was true only in the right hemisphere (*p* < 0.05) for older adults.

**Table 2:** Statistical information for comparing amplitude between the CON condition and the SEM condition in the N400 and P600 time windows.

	N400		P600	
Factor	F	*Post hoc*	*F*	*Post hoc*
C	3.03#	SEM > CON	7.78^**^	SEM > CON
G	0.07		12.84^***^	Y > O
R	0.1		0.04	
H	0.99		1.33	
C × G	13.83^***^	SEM > CON for Y and O	3.60#	SEM > CON for Y and O; Y > O for SEM and CON
R × G	0.12		0.02	
H × G	2.66		1.05	
C × R	3.47#		0.16	
C × H	1.2		0.07	
C × R × G	0.27		5.32^*^	SEM > CON for ANT and POS in both Y and O
C × R × H	6.26^*^		1.12	
C × H × G	0.64		0.65	
R × H × G	0.02		0.11	
C × R × H × G	5.13^*^	ANT and POS: SEM > CON for LH and RH in Y; SEM > CON for RH in O	4.81^*^	Y > O for SEM and CON in ANT, Y > O for SEM in POS

*#*p* < 0.1; ^∗^*p* < 0.05; ^∗∗^*p* < 0.01; ^∗∗∗^*p* < 0.001. C, condition; G, group; R, region; H, hemisphere. Y, younger adults; O, older adults. A, anterior region; P, posterior region; L, left hemisphere; R, right hemisphere.*

**FIGURE 4:**
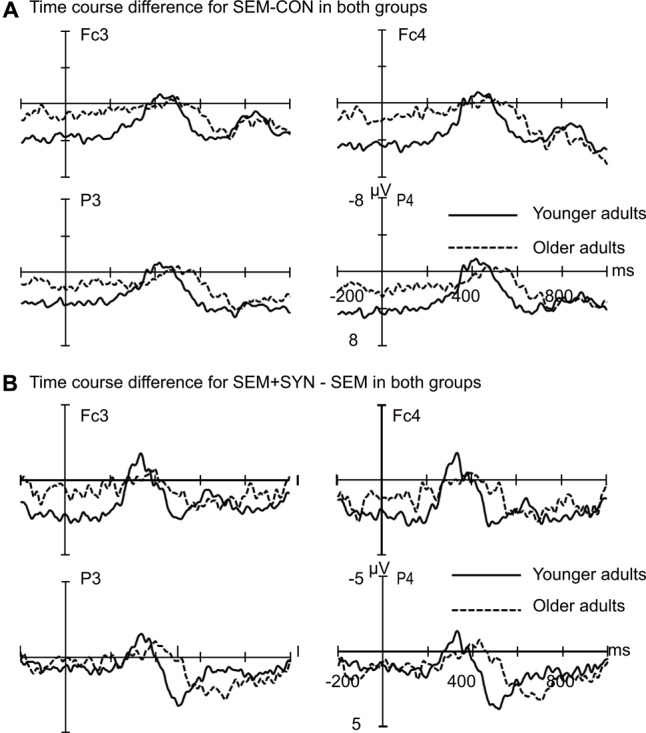
Re-normalized grand averaged ERPs difference between conditions based on the N400 time window for semantic effect and syntactic effect in both groups. **(A)** Grand averaged ERPs difference for semantic effect (SEM – CON) in both younger and older adults. **(B)** Grand averaged ERPs difference for syntactic effect (SEM + SYN – SEM) in both younger and older adults. Re-normalization based on difference in the N400 time window. The solid line represents younger adults and the dashed line represents older adults. Waveforms are time-locked to the onset of the critical words. Negative is plotted upward.

In the P600 time window, the global ANCOVA showed significant main effects of Condition [*F*(1,43) = 7.78, *p* < 0.01] and Group [*F*(1,43) = 12.84, *p* < 0.001], and a marginally significant Group by Condition interaction [*F*(1,43) = 3.60, *p* = 0.06]. Simple main effects revealed that there was a larger P600 amplitude in the SEM condition compared to the CON condition for both younger and older adults (*ps* < 0.001), and a larger P600 amplitude in younger adults compared to older adults for both the SEM (*p* < 0.001) and CON (*p* = 0.03) conditions. In addition, there was a significant Condition by Region by Hemisphere by Group interaction [*F*(1,43) = 4.81, *p* < 0.05] and a significant Condition by Region by Group interaction [*F*(1,43) = 5.32, *p* < 0.05]. Simple main effects revealed a larger P600 amplitude in younger adults compared to older adults in both the SEM (*p* < 0.001) and CON (*p* = 0.02) conditions in the anterior region. In the posterior region, the group difference was found in the SEM condition (*p* < 0.01) but not in the CON (*p* = 0.13) condition. The results mapped to the re-normalized time course profile (**Figure 4A**).

### Syntactic Effect

The statistical results of group amplitude differences are summarized in **Table 3**. **Figure 3A** presents the grand averaged ERPs for the SEM and SEM + SYN conditions, and **Figure 3B** presents the topographic distributions of the difference between the SEM + SYN and SEM conditions in both younger and older adults. In the N400 time window, the global ANCOVA showed no Condition main effect (*F* < 1), Group main effect (*F* < 1), or Group by Condition interaction [*F*(1,43) = 1.43, *p* = 0.24]. However, there was a significant Condition by Region interaction [*F*(1,43) = 3.99, *p* < 0.05], a significant Condition by Region by Group interaction [*F*(1,43) = 7.37, *p* < 0.05], and a significant Condition by Hemisphere by Group interaction [*F*(1,43) = 5.04, *p* < 0.05]. For younger adults, the N400 effect (SEM + SYN > SEM) was found in both anterior and posterior regions and in the left and right hemispheres (*ps* < 0.01). In older adults, the N400 effect was found in the posterior region (*p* = 0.03) but not in the anterior region (*p* = 0.48). There was no significant N400 effect in older adults for either the left (*p* = 0.34) and right (*p* = 0.11) hemisphere.

**Table 3:** Statistical information for comparing amplitude between the SEM condition and the SEM + SYN condition in the N400 and P600 time windows.

	N400		P600	
Factor	*F*	*Post hoc*	*F*	*Post hoc*
C	0.34		2.85#	
G	0.63		5.74^*^	Y > O
R	0.01		0.38	
H	0.06		0.42	
C × G	1.43		1.58	
R × G	0.33		3.60#	A, Y = O; P, Y > O
H × G	2.12		3.37#	
C × R	3.99^*^	A and P, SEM + SYN > SEM	0.09	
C × H	0.92		0.65	
C × R × G	7.37^**^	Y: A and P, SEM + SYN > SEM;	0.05	
		O: P, SEM + SYN > SEM,		
		A, SEM + SYN = SEM		
C × R × H	0.01		2.73	
C × H × G	5.04^*^	Y: L and R, SEM + SYN > SEM;	0.26	
		O: L and R, SEM + SYN = SEM		
R × H × G	0.05		2.62	
C × R × H × G	0.73		3.24#	no G × C interaction in each region

*#*p* < 0.1; ^∗^*p* < 0.05; ^∗∗^*p* < 0.01; ^∗∗∗^*p* < 0.001. C, condition; G, group; R, region; H, hemisphere. Y, younger adults; O, older adults. A, anterior region; P, posterior region; L, left hemisphere; R, right hemisphere.*

In the P600 time window, the global ANCOVA showed a marginally significant main effect of Condition [*F*(1,43) = 2.85, *p* < 0.09; SEM + SYN > SEM] and a significant main effect of Group [*F*(1,43) = 5.74, *p* < 0.05; Younger > Older]. There was a marginally significant Region by Group interaction [*F*(1,43) = 3.60, *p* = 0.06], Hemisphere by Group interaction [*F*(1,43) = 3.60, *p* = 0.06], and Condition by Region by Hemisphere by Group interaction [*F*(1,43) = 3.24, *p* = 0.08]. However, there was no significant Group by Condition interaction in either the left anterior (*p* = 0.18), left posterior (*p* = 0.35), right anterior (*p* = 0.30), or right posterior (*p* = 0.20) regions. The results mapped to the re-normalized time course profile (**Figure 4B**).

### Behavior-ERP Correlations

There was no significant correlation between the semantic effect on accuracy and the N400 effect in either younger (*r* = 0.14, *p* = 0.51) or older adults (*r* = -0.26, *p* = 0.27), nor between the semantic effect on accuracy and the P600 effect in either younger (*r* = 0.34, *p* = 0.14) or older adults (*r* = 0.01, *p* = 0.96). Within each condition, no correlation was found between accuracy and N400 amplitude in either group (*ps* > 0.15). Moreover, no significant correlation was found between posterior P600 amplitude and accuracy in either the CON or the SEM condition (*ps* > 0.10) in younger adults. For older adults, interestingly, there was a correlation between P600 amplitude and accuracy in the SEM (*r* = 0.54, *p* = 0.005) but not the CON condition (*r* = 0.27, *p* = 0.19).

Correlations between the behavioral syntactic effect and the ERP data focused on P600. A larger P600 effect was associated with lower accuracy in the SEM + SYN condition compared to the SEM condition for the older adults (*r* = -0.51, *p* = 0.02) but not for the younger adults (*r* = 0.16, *p* = 0.44). The correlation coefficients were significantly different from each other (Fisher’s *Z* = 2.26, *p* < 0.05). Analyses within each condition revealed a significant correlation between posterior P600 amplitude and accuracy in the SEM (*r* = 0.49, *p* = 0.03) but not the SEM + SYN (*r* = 0.09, *p* = 0.68) condition in younger adults. For older adults, interestingly, there was no correlation between P600 amplitude and accuracy in either the SEM [*r* = -0.06, *p* = 0.82] or the SEM + SYN (*r* = -0.11, *p* = 0.64) condition.

## Discussion

While there is consensus that semantic processing declines with aging, it is an open question whether syntactic processing shows similar declines. In the current study, behavioral analysis revealed a significant age-related decline in both semantic and syntactic processing, indicated by lower accuracy in older adults relative to younger adults during a sentence reading task. Additionally, in the semantic analysis, the older adults showed a smaller amplitude of the N400 effect compared to the younger adults. In the syntactic analysis, while the amplitude of the P600 effect was comparable between groups, correlation analysis revealed that a larger amplitude of the P600 effect was associated with worse syntactic related performance in older adults only. Whereas previous studies mainly found an age-related decline in comprehending complex sentences, the present results suggest that there is also a decline in neural efficiency, seen in errors during syntactic processing of canonical Chinese sentences with simple structures. The present study is the most well-controlled study to date to document age-related declines in syntactic processing.

### Semantic Processing

The age-related decline in semantic processing observed in the present study was in line with previous ERP studies (Federmeier and Kutas, 2005; Federmeier et al., 2010; DeLong et al., 2012) and fMRI studies (Grossman et al., 2002a,b). Moreover, previous research found delayed N400 peak latency in older adults for sentence-level congruency (Federmeier et al., 2003, 2010), consistent with the current delayed N400 effect illustrated in the time course and topographic maps (**Figure 2**). The reduced N400 effect in older adults has been linked to less efficiency in pre-activating semantic features of coming words (Federmeier and Kutas, 2005; Federmeier et al., 2010; DeLong et al., 2012) and integrating the words into context (Hagoort et al., 2004; Zhu et al., 2012). Similarly, fMRI studies of older adults have revealed under-recruitment in the left frontal-temporal regions in poorer readers compared to better readers (Grossman et al., 2002b) or in older adults compared to younger adults (Zhu et al., 2017). The reduction of the N400 effect, however, was not correlated with accuracy differences between conditions in either group. Therefore, the correlation pattern may suggest a dedifferentiation between congruent and incongruent sentences in older adults (Li et al., 2001). The older adults were less able to predict the coming words and/or they exerted less effort to integrate the words into context, resulting in delayed peak latency and reduced amplitude for the N400. In contrast to the preserved auditory comprehension at a normal rate, the present study found that declined comprehension may be related to slightly higher task demand in a reading than listening task (Diaz et al., 2016). Such speculation could be tested in future studies.

Unlike the N400 effect, which was clearly present in both groups, the semantic P600 effect was seen only in older adults, which is in line with our recent finding in a different sample (Xu et al., 2017). After re-normalization, the amplitude of the semantic P600 effect was also reduced in the older adults relative to the younger adults during semantic processing. The semantic P600, also called late positive complex in some studies, has been used as an index of reanalysis of semantic information, or the cost of failure to predict upcoming words (reviewed in Van Petten and Luka, 2012).

Combining the amplitude change and behavior-ERP correlation may help to clarify the functional significance of the P600. Although there was no behavior-ERP correlation for the semantic effect in either group, higher P600 amplitude was correlated with higher accuracy in the SEM condition in both groups. Given that the older adults showed lower accuracy in the SEM condition than the younger adults, one may argue that older adults may try to reinterpret the implausible sentence, making it harder to reject, in contrast to the younger adults. If this inference is true, then more reinterpretation may be associated with lower accuracy, which was the opposite of the present behavior-ERP correlation result. Moreover, in a recent study with a similar design, we did not find evidence that the P600 was related to semantic prediction cost in the older adults (Xu et al., 2017). For both younger and older adults, larger P600 amplitude was associated with higher accuracy in the SEM condition, suggesting that, like the younger adults, the older adults were able to modulate the brain in semantic integration. Such a modulation effect is partly in line with previous findings indicating that older adults showed successful compensation (Peelle et al., 2010) or preserved semantic processing (Madden and Dijkstra, 2010). Nonetheless, the significant P600 effect reduction in the older adults also suggests a decline in the discrimination of semantic congruency.

### Syntactic Processing

Based on the behavioral data, we found worse syntactic processing in the older adults compared to the younger adults. It should be noted that in the current study, the measure of performance was accuracy rather than reaction time (RT). It has been argued that slowed RT could be due to general cognitive slowing rather than specific slowing in syntactic processing (Salthouse, 1996; Kemmer et al., 2004). However, the accuracy in comprehension cannot be solely explained by processing speed. Performance is one of the criteria in judging whether there is preserved or declined syntactic processing in aging. In previous studies, it was debated whether syntactic processing declined or was preserved in aging. While comparable performance in younger and older adults was found in some studies (Tyler et al., 2010; Campbell et al., 2016), worse performance in older adults was also observed in other studies (Kemmer et al., 2004; Kemper and Herman, 2006; Peelle et al., 2010). Interestingly, while Kemmer et al. (2004) claimed that older adults preserved syntactic processing, older adults’ accuracy was significantly lower than the younger adults’ in a comprehension task. The worse performance observed in the older adults in the current study demonstrated a decline in syntactic processing in our aging sample.

ERP data could provide further evidence of a neural mechanism that would explain the preservation or decline in behavioral performance. In the current study, the ERP results revealed delayed peak latency for both the N400 and P600 effect in older adults. Moreover, in comparisons between the SEM + SYN condition and the SEM condition, there was a smaller N400 effect in older compared to younger adults and a comparable P600 effect between age groups. Normally Chinese sentence comprehension tasks generate a larger N400 amplitude in the SEM + SYN condition relative to the SEM condition (Yu and Zhang, 2008; Zhang et al., 2013) because syntactic violation always accompanies semantic violation in Chinese sentences (Wang et al., 2008). In the current study, the age-related N400 reduction in the older adults suggests a decline in line with semantic processing.

The key finding of the present study was that syntactic processing was less efficient in older adults. The P600 effect for syntactic processing cannot be attributed to working memory, verbal intelligence, or fluency, as these factors were well matched between groups in the present study. Moreover, the present study employed canonical BA and BEI construction sentences; these sentences were much simpler than sentences used in previous research, which had a complex relative clause (Grossman et al., 2002a; Kemmer et al., 2004). Such a P600 effect pattern suggests that the older adults were able to respond to the syntactic violation as younger adults did (Kemmer et al., 2004; Tyler et al., 2010). While previous research has also found a P600 of equal amplitude between age groups (Kemmer et al., 2004), the functional significance of the P600 remains unclear.

While the older adults performed better in the SEM + SYN condition than in the SEM condition, they performed worse than the younger adults. It is possible that the accuracy reflected a task demand effect, given that the same sentences could be harder for older adults than for younger adults. However, the worse performance should be explained with behavior-ERP correlation. Critically, behavior-ERP correlation analysis revealed that the larger P600 effect was linked with less accuracy in the SEM + SYN condition compared to the SEM condition. Moreover, the peak latency of the P600 was significantly later for the older adults than for the younger adults, regardless of correction for the N400 peak latency. The correlation pattern may suggest that the older adults were able to modulate their neural response when the task demands increased (Geerligs et al., 2014). Moreover, the correlation results may also suggest that the P600 effect in the older adults reflected a less efficient response (Zhu et al., 2015). That is, perhaps the larger P600 was found in older adults because they failed to effectively use syntactic information during reading.

Consistent with these possibilities, a less effective neural response in older adults was also found in previous studies (Colcombe et al., 2005; Zarahn et al., 2007; Stern, 2009). For instance, age-related over-recruitment in the frontal-parietal network was associated with worse performance in a cognitive control task (Zhu et al., 2015). Such findings support our correlation results. However, we should be cautious in aligning results from ERPs studies and those from fMRI studies. In fact, age-related over-recruitment and under-recruitment can be found in the same participants (Grossman et al., 2002b; Peelle et al., 2010; Erb and Obleser, 2013). As most previous studies have employed an fMRI technique, the relationship between ERP results and fMRI results requires further investigation. Compared with the low temporal resolution in both behavioral and fMRI measurement, however, the high temporal resolution of the ERP technique helps us to capture cognitive processes that happen quickly. The behavior-ERP correlation, which integrated on-line and off-line information, indicates a trend of age-related decline in syntactic processing.

Moreover, the less efficient neural response observed here is not necessarily contradictory to previous fMRI studies, which have found compensatory response (Peelle et al., 2010) or maintained response (Campbell et al., 2016) in the brain activity for syntactic processing. It is possible that different types of mechanisms, including both less efficient and compensatory mechanisms, could exist in the same participants (Logan et al., 2002; Peelle et al., 2010; Hakun et al., 2015).

One may ask what exact type of processing was indexed by P600, given that both semantic and syntactic violation induced a P600 amplitude increase. While one could not infer the presence of syntactic processing based on the presence of P600 (Poldrack, 2006), the manipulation in the present study included syntactic violation. Specifically, the BA and BEI sentences show canonical syntactic construction, which requires a specific verb after the second noun phrase. A noun rather than a verb presented in the verb place significantly violated the syntactic rules. The violation, compared to the violation in the SEM sentence, induced a significantly larger P600 amplitude. Therefore, we infer syntactic disruption related processes during the P600 time window. The exact processes may include but are not limited to the following: categorical violation, disruption of argument structure building, and/or failure in syntactic integration (Hagoort et al., 1993; Friederici, 2002; Bornkessel and Schlesewsky, 2006). Because the syntactic clue highly depends on sentence context, the violation of syntactic information is typically in a later time window, such as the P600 (Zhou et al., 2010; Jiang and Zhou, 2012; Wang et al., 2012; Zhang et al., 2013; Yang et al., 2015).

There were limitations of the present study that should be noted. First, as previous studies consistently found age-related alterations in brain structure (Raz et al., 2005, 2007; Madden et al., 2009), it would be interesting to test whether brain volume, cortical shrinking or white matter integrity contribute to syntactic decline (Madden et al., 2009). Moreover, because functional network maintenance has been found to be important for syntactic processing in older adults (Campbell et al., 2016), it is also important to test the relationship between brain function and structural change (Tremblay et al., 2013).

## Conclusion

The aim of the present study was to test whether there was an age-related decline in syntactic processing, as has been found for semantic processing. The pattern of behavioral and ERP results during a sentence comprehension task suggested a decline in both types of processing, with a trend in the data suggesting that syntactic processing is also less efficient with age. The study design allows strong inferences about causality, as the older and younger groups were matched on general intelligence, verbal intelligence, fluency and working memory. The present study is the first to document that syntactic processing declines during aging.

## Ethics Statement

This study was carried out in accordance with the recommendations of Ethic Committee of Jiangsu Normal University with written informed consent from all subjects. All subjects gave written informed consent in accordance with the Declaration of Helsinki. The protocol was approved by the Ethic Committee of Jiangsu Normal University.

## Author Contributions

ZZ and YY conceived and designed the experiment, wrote the manuscript, and approved the final version of the manuscript. XH prepared the stimuli and collected the data. ZZ and XH analyzed the data.

## Conflict of Interest Statement

The authors declare that the research was conducted in the absence of any commercial or financial relationships that could be construed as a potential conflict of interest.

## Footnotes

1 http://www.mocatest.org/
